# Closed-Loop Iterative Self-Calibration of Initial Phase in Phased Arrays

**DOI:** 10.3390/s26103201

**Published:** 2026-05-19

**Authors:** Xinyu Huang, Deshun Huang, Bingbing Chen, Bo Wu

**Affiliations:** 1 School of Electronic and Information Engineering, Anhui University, Hefei 230601, China; p24201027@stu.ahu.edu.cn (X.H.); hds25053@163.com (D.H.); 2 School of Electronic Information and Integrated Circuits, Hefei Normal University, Hefei 230601, China; zcbb_2011@163.com

**Keywords:** beamforming, closed-loop iteration, initial phase calibration, phase error compensation, phased array antenna

## Abstract

This paper proposes a closed-loop iterative automatic initial phase calibration scheme. Under the condition that the direction of the calibration source and the signal frequency are known, the complex readback data of all array elements are obtained. The theoretical phase of each array element is calculated in advance and removed. Then, conjugate multiplication with a reference element is performed to eliminate the common phase error and construct the relative phase residual. Finally, the relative phase residual is fed back to the phase compensation table and iteratively updated, thereby achieving rapid automatic calibration of the phased array. The simulation results show that, for a 256-element array, the digital computation time of the proposed method is approximately 1.6 ms, excluding signal acquisition and readback time, and the phase compensation table can converge rapidly. Based on the simulated results and normalized comparison of the array factors before and after calibration, the peak response in the target main-lobe direction is improved by approximately 14.69 dB, while the highest side-lobe level relative to the main lobe is further reduced by approximately 12.72 dB. These results demonstrate that the proposed algorithm can effectively improve beam focusing performance and side-lobe suppression, providing a new implementation scheme for initial phase calibration of large-scale phased arrays.

## 1. Introduction

With the rapid development of satellite internet and 6G integrated space–air–ground communication technologies, Low Earth Orbit (LEO) satellite constellations are becoming an important foundation for future wireless communication systems [1,2,3]. Meanwhile, increasingly complex and dynamic wireless communication scenarios have imposed higher requirements on the beam control capability and adaptive performance of antenna systems. Owing to their advantages of flexible beamforming, accurate beam steering, and fast scanning speed, phased array antennas have attracted extensive attention from researchers. They have been widely applied in satellite internet systems [4,5], radar sensing [6,7,8], and autonomous driving [9,10]. However, the beam control performance of phased array antennas relies on the consistency of the initial phases among all array elements. Once phase deviations occur, problems such as beam pointing offset, main-lobe distortion, and side-lobe level increase may arise. Therefore, initial phase calibration is crucial for ensuring the performance of phased array antennas [11,12].

On the one hand, existing initial phase calibration methods mainly include the near-field scanning method and the Rotating Element Vector (REV) method. The near-field scanning method mainly relies on the mechanical movement of a probe to perform point-by-point scanning. Although it offers high calibration accuracy, it requires sampling over the entire spatial grid, resulting in long measurement time, which becomes particularly significant for large-scale phased arrays [13]. To address this issue, ref. [14] proposed a parallel probe sampling method by designing an irregular probe array and optimizing the continuous scanning sequence, thereby significantly improving the testing speed over conventional near-field scanning while keeping the measurement error below 5%. In addition, ref. [15] proposed a continuous implementation of near-field scanning, in which measurements are carried out during probe motion, thus avoiding point-by-point stopping and significantly reducing the calibration time. Although these optimization schemes effectively shorten the measurement time, they still cannot fundamentally eliminate the dependence on mechanical scanning structures. To overcome this limitation, researchers proposed the rotating element vector (REV) method, in which the phase of each array element is rotated individually and the received power at an external observation point is measured as a function of the element phase [16], thereby enabling rapid calibration and compensation of the array. The REV method does not rely on probe movement; however, for large-scale phased arrays, the phase variation in a single element causes only very slight fluctuations in the overall array power, making the power curve flatter and thus reducing system robustness. To address this issue, ref. [17] divided the array elements into several groups and performed synchronized phase rotation within each group, thereby producing more pronounced power variations and effectively improving the robustness of the initial phase calibration method. In addition, the REV method places stringent requirements on phase-shifter accuracy. To solve this problem, ref. [18] proposed the rotating harmonic electric-field vector (RHEV) method, which only requires one-bit phase shifters (0°/180°). By using an FPGA to precisely control the periodic phase switching of each channel, the requirement on phase-shifter resolution is substantially relaxed.

On the other hand, recent studies have begun to investigate calibration frameworks based on complex array-signal measurement and over-the-air (OTA) calibration. For example, ref. [19] used the measured received power as the objective function and employed the simultaneous perturbation stochastic gradient descent (SPGD) algorithm for optimization. Random phase perturbations were simultaneously applied to all array elements, followed by gradient-based iterative updating, thereby achieving efficient phase calibration. In addition, ref. [20] proposed a direct linear-equation solution method based on complex signal measurement. In this method, the beam direction of the entire array is frequently adjusted during measurement. For the complex signals received by a far-field probe, a beam-angle selection strategy is introduced to reduce the condition number of the matrix, after which matrix inversion is used to accurately solve for the initial phase errors of all array elements, achieving high-precision phase calibration with very few measurements.

In summary, although existing methods are capable of initial phase calibration for phased array antennas, their practical implementation is still constrained by factors such as mechanical structures, computational complexity, and array scale. To this end, this paper proposes a fast closed-loop iterative automatic phase calibration method for phased array systems. By transmitting an external calibration signal and collecting the responses of all array elements under known incident angle and signal frequency conditions, the proposed method constructs relative phase residuals and updates the phase compensation table in real time, thereby enabling automatic phase calibration without mechanical scanning, without element-by-element phase sweeping, and with fast convergence.

## 2. Algorithm

### 2.1. Model

**Figure 1:**
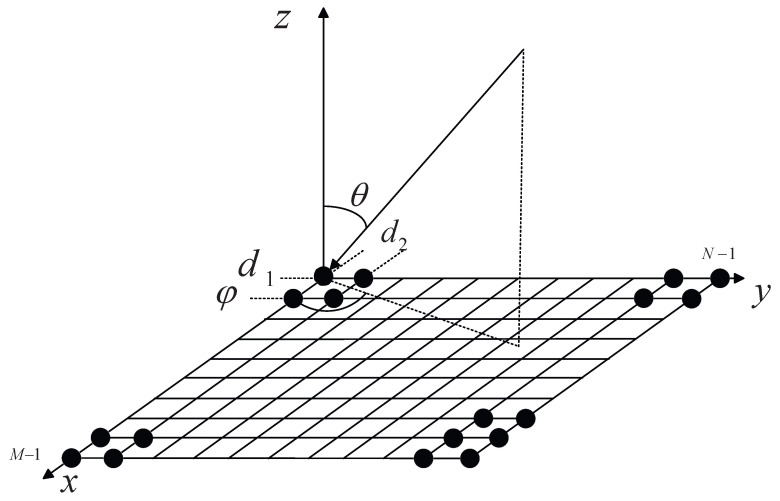
Incident diagram of the planar phased array.

As shown in Figure 1, for an M×N large-scale planar phased array, the antenna elements are located on the XOY plane, with element spacings of $d_{1}$ and $d_{2}$ in the *X* and *Y* directions, respectively.

Assume that a narrowband far-field signal is incident from the direction (θ,φ); then, the direction cosines can be expressed as follows:

(1) u=sinθcosφ,v=sinθsinφ.

Then, the theoretical phase $\varphi_{\mathrm{th}}\left(m,n\right)$ of the (m,n)th array element (m=0,…,M−1, n=0,…,N−1) with respect to the coordinate origin can be expressed as follows:

(2) $$\varphi_{\mathrm{th}}\left(m,n\right)=k_{0}\left(md_{1}u+nd_{2}v\right),$$

where $k_{0}=\frac{2\pi }{\lambda }$. At the *k*th snapshot, the theoretical received signal of the (m,n)th array element can be expressed as follows:

(3) $$x^{\left(k\right)}\left(m,n\right)=s^{\left(k\right)}e^{j\varphi_{\mathrm{th}}\left(m,n\right)}.$$

where k=1,2,…,K is the snapshot index, and $s^{\left(k\right)}$ is the incident narrowband signal at the *k*th snapshot. It is assumed that the incident signal remains unchanged over all snapshots, that is, $s^{\left(k\right)}=s$.

### 2.2. Algorithm Model

Under ideal conditions, the phase response of the signal received by each array element is jointly determined by the geometric position of the element, the signal incident direction, and the signal frequency. However, in practical engineering applications, due to factors such as hardware manufacturing errors and device aging, the initial phases of different channels are inconsistent. Therefore, initial phase calibration is required, and its core objective is to make the initial phases of all array elements consistent.

Assuming that the incident direction and signal frequency of the incident narrowband far-field signal *s* are known, the received signal of element (m,n) at the *k*th snapshot can be modeled as follows:

(4) $$y^{\left(k\right)}\left(m,n\right)=se^{j\varphi_{th}\left(m,n\right)}e^{j\varphi_{com}\left(k\right)}e^{j\psi (m,n)}+w^{\left(k\right)}\left(m,n\right)$$

where $e^{j\varphi_{th}\left(m,n\right)}$, $e^{j\varphi_{com}\left(k\right)}$, and $e^{j\psi (m,n)}$ represent the theoretical phase, the common phase, and the initial phase of the array element, respectively. $w^{\left(k\right)}\left(m,n\right)$ represents the receiver noise of the system. The initial phase of the array element, $e^{j\psi (m,n)}$, is the calibration target, while the common phase $e^{j\varphi_{com}\left(k\right)}$ represents the overall phase perturbation that is approximately identical for all array elements during sampling at the *k*th snapshot.

#### 2.2.1. Removal of Theoretical Phase

Assuming that the incident direction and signal frequency of the incident narrowband far-field signal *s* are known, the theoretical phase $e^{j\varphi_{th}\left(m,n\right)}$ of the (m,n)th array element can be determined by the array geometry and the incident direction, and can be calculated from Equation (2).

First, the theoretical phase corresponding to each array element is calculated, and the received complex signal is multiplied by $e^{-j\varphi_{th}\left(m,n\right)}$ to eliminate the theoretical phase term, that is:

(5) $$Z^{\left(k\right)}\left(m,n\right)=y^{\left(k\right)}\left(m,n\right)\;e^{-j\;\varphi_{\mathrm{th}}\left(m,n\right)}$$

$Z^{\left(k\right)}\left(m,n\right)$ contains the initial phase, the common phase, and the noise term of the (m,n)th array element.

#### 2.2.2. Removal of Common Phase and Construction of Relative Phase Residual

To eliminate the common phase of the system and extract the relative phase residual of each array element with respect to the reference element, the $\left(m_{0},n_{0}\right)$th array element is selected as the phase reference. The signal of each array element after theoretical phase removal is then multiplied by the complex conjugate of the corresponding signal of the reference element. In this way, the common phase within the same snapshot is canceled, leaving only the relative phase residual between the calibration target element (m,n) and the reference element $\left(m_{0},n_{0}\right)$, as well as the noise term, i.e.:

(6) $$r^{\left(k\right)}\left(m,n\right)=Z^{\left(k\right)}\left(m,n\right)\;Z^{(k)\;*}\left(m_{0},n_{0}\right).$$

To reduce the influence of random noise on phase estimation, cumulative averaging is performed on $r^{\left(k\right)}$ over *K* snapshots, namely:

(7) $$\bar{r}\left(m,n\right)=\frac{1}{K}\sum_{k=1}^{K}r^{\left(k\right)}\left(m,n\right)$$

### 2.3. Initial Phase Calibration Algorithm Based on Closed-Loop Iteration

The main function of multi-snapshot averaging is to suppress random sampling noise. However, there still exist errors caused by phase shifter control errors and changes in the array operating state after the compensation is applied, making it difficult for a single multi-snapshot observation to guarantee the final calibration accuracy. Therefore, on the basis of multi-snapshot observation, this paper introduces a closed-loop iterative mechanism. Through the process of “observation–compensation–re-observation”, the system is corrected round by round so that it approaches the optimal operating state.

Denote the observed phase residual of the (m,n)th array element at the *t*th iteration as $\Delta \phi^{\left(t\right)}\left(m,n\right)$, which represents the phase difference between the calibration target array element (m,n) and the reference array element $\left(m_{0},n_{0}\right)$. The observed residual phase can be obtained by taking the angle of $\bar{r}\left(m,n\right)$. In order to facilitate the round-by-round correction of the phase error of each array element, this paper introduces a phase compensation table to record the phase compensation applied to each array element at each iteration. By feeding the current $\Delta \phi^{\left(t\right)}\left(m,n\right)$ back to the input and continuously updating the compensation table in a recursive manner, a fast phase calibration algorithm based on closed-loop iteration is constructed. The specific process is as follows:

#### 2.3.1. Pre-Compensation Operation

First, through pre-compensation processing, a coarse correction of the initial phase error of the array elements can be performed before the iteration starts, thereby effectively reducing the initial phase error of the array elements and accelerating the convergence speed of the system. During the initial observation, the initial value of the compensation table is set to 0; that is, no additional phase compensation is applied to each array element. Under this condition, the array readback signals are acquired. $\Delta \phi^{\left(0\right)}\left(m,n\right)$ denotes as the initial phase value, and then the negative of the initial phase value is written into the phase compensation table as the initial value for the subsequent closed-loop iteration, namely:

(8) $$\phi_{\mathrm{comp}}^{\left(0\right)}\left(m,n\right)=-\Delta \phi^{\left(0\right)}\left(m,n\right).$$

#### 2.3.2. Closed-Loop Iteration

Then, closed-loop iteration is performed and the compensation table is updated. The iteration process can be expressed as follows:

(9) $$\phi_{\mathrm{comp}}^{\left(t\right)}\left(m,n\right)=\phi_{\mathrm{comp}}^{\left(t-1\right)}\left(m,n\right)-\mu \Delta \phi^{\left(t\right)}\left(m,n\right).$$

where μ is the iteration step size, and its value range is 0<μ<1.

#### 2.3.3. Iteration Termination Condition

In this paper, the maximum absolute value of the observed phase residual is used as the stopping criterion, namely:

(10) $$\Delta \phi_{\max}^{\left(t\right)}=\max_{m,n}\left|\Delta \phi^{\left(t\right)}\left(m,n\right)\right|.$$

When $\Delta \phi_{\max}^{\left(t\right)}<\tau$ or $t=T_{\max}$, the iteration terminates. The flow chart of the algorithm is shown in Algorithm 1.

### 2.4. Noise Analysis

In an actual system, the true phase residual cannot be obtained directly, and only its observed value can be acquired through collected data. As defined previously, the observed residual phase at the *t*th iteration is $\Delta \phi^{\left(t\right)}\left(m,n\right)$, namely:

(11) $$\Delta \phi^{\left(t\right)}\left(m,n\right)\approx \varepsilon^{\left(t\right)}\left(m,n\right)+\eta^{\left(t\right)}\left(m,n\right),$$

where $\eta^{\left(t\right)}\left(m,n\right)$ represents the observation noise of the collected data at the *t*th iteration. $\varepsilon^{\left(t\right)}\left(m,n\right)$ represents the true residual phase of element (m,n) relative to the reference element $\left(m_{0},n_{0}\right)$ at the *t*th iteration.

To further analyze the influence of noise on the closed-loop iterative calibration process, the fixed true phase difference to be compensated in the system is defined. Denote the true hardware phase difference in the (m,n)th array element relative to the reference array element $\left(m_{0},n_{0}\right)$ as follows:

(12) $$\Delta \psi \left(m,n\right)=\psi \left(m,n\right)-\psi \left(m_{0},n_{0}\right).$$

where Δψ(m,n) is the fixed true hardware phase difference in the array element (m,n) relative to the reference array element $\left(m_{0},n_{0}\right)$, and it is the true object to be compensated in the system.
**Algorithm 1** Initial Phase Calibration Algorithm Based on Closed-Loop Iteration**Require:** Array size (M,N); reference element $\left(m_{0},n_{0}\right)$; number of snapshots *K*; iteration step size μ; threshold τ; maximum number of updates $T_{\max}$
**Ensure:** Final phase compensation table $\phi_{\mathrm{comp}}\left(m,n\right)$
      **Initial observation and pre-compensation**
  1: **for**
k=1 to *K* **do**
  2:      Acquire the *k*th snapshot data $y_{k}\left(m,n\right)$
  3:      Remove the theoretical phase according to (5) to obtain $Z_{k}\left(m,n\right)$
  4:      Construct the reference conjugate product $r_{k}\left(m,n\right)$ according to (6)
  5: **end for**
  6: $\bar{r}\left(m,n\right)\leftarrow \frac{1}{K}\sum_{k=1}^{K}r_{k}\left(m,n\right)$
  7: $\Delta \phi^{\left(0\right)}\left(m,n\right)\leftarrow ∠\;\left(\bar{r}\left(m,n\right)\right)$
  8: Generate the pre-compensation table according to (8):
  9: $\phi_{\mathrm{comp}}^{\left(0\right)}\left(m,n\right)\leftarrow -\Delta \phi^{\left(0\right)}\left(m,n\right)$
      **Closed-loop iterative update**
10: **for**
t=1 to $T_{\max}$ **do**
11:      Apply the compensation table $\phi_{\mathrm{comp}}^{\left(t-1\right)}\left(m,n\right)$
12:      **for** k=1 to *K* **do**
13:            Acquire the *k*th snapshot data $y_{k}^{\left(t\right)}\left(m,n\right)$
14:            Remove the theoretical phase according to (5) to obtain $Z_{k}^{\left(t\right)}\left(m,n\right)$
15:            Construct the reference conjugate product $r_{k}^{\left(t\right)}\left(m,n\right)$ according to (6)
16:      **end for**
17:      ${\bar{r}}^{\left(t\right)}\left(m,n\right)\leftarrow \frac{1}{K}\sum_{k=1}^{K}r_{k}^{\left(t\right)}\left(m,n\right)$
18:      $\Delta \phi^{\left(t\right)}\left(m,n\right)\leftarrow ∠\;\left({\bar{r}}^{\left(t\right)}\left(m,n\right)\right)$
19:      **if** $\max_{m,n}\left|\Delta \phi^{\left(t\right)}\left(m,n\right)\right|<\tau$ **then**
20:            **return** $\phi_{\mathrm{comp}}^{\left(t-1\right)}\left(m,n\right)$
21:      **end if**
22:      $\phi_{\mathrm{comp}}^{\left(t\right)}\left(m,n\right)\leftarrow \phi_{\mathrm{comp}}^{\left(t-1\right)}\left(m,n\right)-\mu \Delta \phi^{\left(t\right)}\left(m,n\right)$
23: **end for**
24: **return**
$\phi_{\mathrm{comp}}\left(m,n\right)$


According to the algorithm flow shown in Algorithm 1, before the *t*th round of closed-loop observation, the compensation table actually issued by the system is $\phi_{\mathrm{comp}}^{\left(t-1\right)}\left(m,n\right)$. Therefore, the true residual phase corresponding to the *t*th round of observation can be expressed as follows:

(13) $$\varepsilon^{\left(t\right)}\left(m,n\right)=\Delta \psi \left(m,n\right)+\phi_{\mathrm{comp}}^{\left(t-1\right)}\left(m,n\right),\;t\geq 1.$$

After the *t*th round of observation is completed, the compensation table is updated as follows:

(14) $$\phi_{\mathrm{comp}}^{\left(t\right)}\left(m,n\right)=\phi_{\mathrm{comp}}^{\left(t-1\right)}\left(m,n\right)-\mu \Delta \phi^{\left(t\right)}\left(m,n\right).$$

Therefore, the true residual phase corresponding to the (t+1)th round of observation is:

(15) $$\begin{array}{cc} \varepsilon^{\left(t+1\right)}\left(m,n\right) & =\Delta \psi \left(m,n\right)+\phi_{\mathrm{comp}}^{\left(t\right)}\left(m,n\right) \end{array}$$

(16) $$\;\;\;\;\;\;\;\;\;\;\;\;\;\;\;\;\;=\Delta \psi \left(m,n\right)+\phi_{\mathrm{comp}}^{\left(t-1\right)}\left(m,n\right)-\mu \Delta \phi^{\left(t\right)}\left(m,n\right)$$

(17) $$\;\;\;\;\;\;\;\;=\varepsilon^{\left(t\right)}\left(m,n\right)-\mu \Delta \phi^{\left(t\right)}\left(m,n\right).$$

Substituting (11) into the above equation, the recursive relationship of the true residual phase is obtained as follows:

(18) $$\begin{array}{cc} \varepsilon^{\left(t+1\right)}\left(m,n\right) & \approx \varepsilon^{\left(t\right)}\left(m,n\right)-\mu \left[\varepsilon^{\left(t\right)}\left(m,n\right)+\eta^{\left(t\right)}\left(m,n\right)\right] \end{array}$$

(19) $$\;\;\;=\left(1-\mu \right)\varepsilon^{\left(t\right)}\left(m,n\right)-\mu \eta^{\left(t\right)}\left(m,n\right).$$

It can thus be seen that the true residual phase after closed-loop iteration, $\varepsilon^{\left(t+1\right)}\left(m,n\right)$, is jointly determined by the true residual phase of the previous round, $\varepsilon^{\left(t\right)}\left(m,n\right)$, and the current observation noise term, $\eta^{\left(t\right)}\left(m,n\right)$. (1−μ) determines the convergence speed of the residual, while $-\mu \eta^{\left(t\right)}\left(m,n\right)$ indicates that the observation noise will be introduced into the compensation table during each round of update. Therefore, in practical applications, μ can be adjusted according to the SNR to balance the calibration accuracy and calibration speed.

## 3. Comparison of Algorithm Performance

### 3.1. Comparison with Existing Methods

This section compares the calibration time of the near-field scanning method, the rotating element vector (REV) method, and the closed-loop iterative automatic calibration method proposed in this paper. The calibration time can be divided into the data acquisition time $T_{col}$ and the computation time $T_{cal}$, namely:

(20) $$T_{\mathrm{total}}=T_{col}+T_{cal}$$

Assume that the total number of array elements is MN, that *K* snapshots are acquired each time, that the sum of the preprocessing and iteration numbers is $N_{it}$, and that the sampling time is denoted by $T_{a}$. Then, the data acquisition time can be expressed as follows:

(21) $$T_{col,\mathrm{prop}}=N_{it}KT_{a}.$$

The near-field scanning method usually requires the probe to move along the grid. Assume that the number of grid points is *G*, that the probe movement and stabilization time is $T_{move}$, and that the acquisition time is $T_{\mathrm{samp}}$. Then, the sampling time can be approximately expressed as follows:

(22) $$T_{col,\mathrm{NF}}\approx G\left(T_{\mathrm{move}}+T_{\mathrm{samp}}\right).$$

The ratio of the required data acquisition times is given as follows:

(23) $$\frac{T_{col,\mathrm{NF}}}{T_{col,\mathrm{prop}}}\approx \frac{G\left(T_{\mathrm{move}}+T_{\mathrm{samp}}\right)}{N_{\mathrm{it}}\;K\;T_{a}}.$$

Since the probe adopts mechanical movement, its stabilization time $T_{\mathrm{move}}$ is usually on the order of seconds, which is much larger than the digital sampling time $T_{a}$ in the proposed method (i.e., $T_{\mathrm{move}}\gg T_{a}$). Therefore, the closed-loop iterative method proposed in this paper is faster than the near-field scanning method in terms of calibration time.

For the REV method, the data acquisition process requires applying *P* phase states to the array element to be calibrated and observing the corresponding output power. Let $T_{ps}$ denote the time required to change the phase, and let $T_{sw}$ denote the sampling time. Then, the data acquisition time can be expressed as follows:

(24) $$T_{col}=\left(MN-1\right)P\left(T_{ps}+T_{sw}\right)$$

Therefore, the ratio of the data acquisition time of the REV method to that of the proposed method is:

(25) $$\frac{T_{col,\mathrm{REV}}}{T_{col,\mathrm{prop}}}=\frac{\left(MN-1\right)P\left(T_{ps}+T_{sw}\right)}{N_{it}KT_{a}}.$$

It can be seen from the above equation that the data acquisition time of the REV method is linearly related to the number of array elements MN and the number of phase states *P*, whereas the data acquisition time of the proposed method depends on the single array observation time $T_{a}$, the number of snapshots *K*, and the number of iterations $N_{it}$. Unlike the REV method, which requires multiple phase states to be applied to each array element to be calibrated one by one, the proposed method does not require element-by-element phase adjustment testing, and its data acquisition procedure is more concise.

In terms of data computation, the computational complexity of REV for solving each array element can be approximately expressed as O(MNP), whereas the computational complexity of the proposed method can be approximately expressed as $O\;\left(MN\;N_{\mathrm{it}}\;K\right)$, where the computation load mainly depends on the number of iterations and the number of snapshots. However, the calibration procedure of REV is an open-loop calibration. In contrast, the proposed method is a closed-loop calibration, which can fully utilize the array information of *K* snapshots for error convergence. This closed-loop mechanism significantly improves the robustness of the algorithm under low signal-to-noise ratio conditions and is more suitable for the online dynamic calibration requirements of large-scale phased arrays.

### 3.2. Comparison with Emerging Methods

This subsection further compares the proposed closed-loop iterative automatic calibration method with over-the-air phased array calibration based on the SPGD algorithm [19] and deep-learning-based phase calibration [21].

The method proposed in [19] is a random optimization calibration approach based on power feedback. In each iteration, random perturbations are simultaneously applied to all array elements, and the array phase compensation is updated according to the power measurement results.

The method proposed in [21] learns the mapping relationship among the target beam direction, the theoretical phase, and the phase correction. It takes the target beam angle and the theoretical phase as inputs, uses approximately 8000 HFSS full-wave simulations to generate the training dataset, and learns to predict the correction required for the theoretical phase.

To more clearly illustrate the differences among these methods, Table 1 presents a comparison between them and the proposed method.

## 4. Algorithm Performance Analysis

This section analyzes the proposed algorithm from three aspects: noise robustness, convergence characteristics, and computation time. The Root Mean Square Error (RMSE) is introduced as the evaluation metric. The residual RMSE of the array at the *t*th iteration is defined as follows:

(26) $${\mathrm{RMSE}}^{\left(t\right)}=\sqrt{\frac{1}{MN}\sum_{m=1}^{M}\sum_{n=1}^{N}{\left(\Delta \phi_{m,n}^{\left(t\right)}\right)}^{2}}$$

### 4.1. Noise Robustness

**Figure 2:**
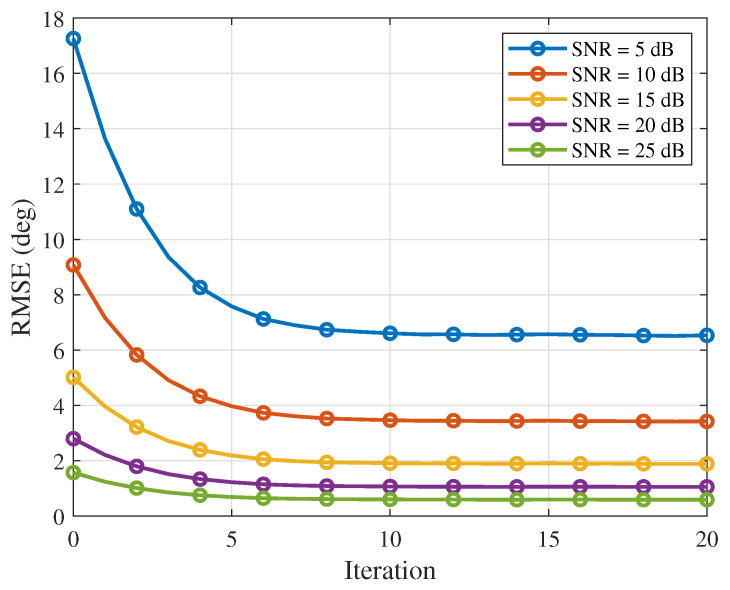
RMSE versus the number of iterations (SNR = 5 dB, 10 dB, 15 dB, 20 dB and 25 dB).

After fixing the iteration step size and the number of snapshots (μ = 0.25, *K* = 4), the variation in the RMSE of the proposed algorithm with the number of iterations when the SNR is 5 dB, 10 dB, 15 dB, 20 dB and 25 dB is shown in Figure 2. The simulation shows that, as the number of iterations increases, the RMSE of the proposed algorithm gradually decreases; as the SNR increases, the RMSE gradually decreases. The simulation results are consistent with the theoretical analysis.

**Figure 3:**
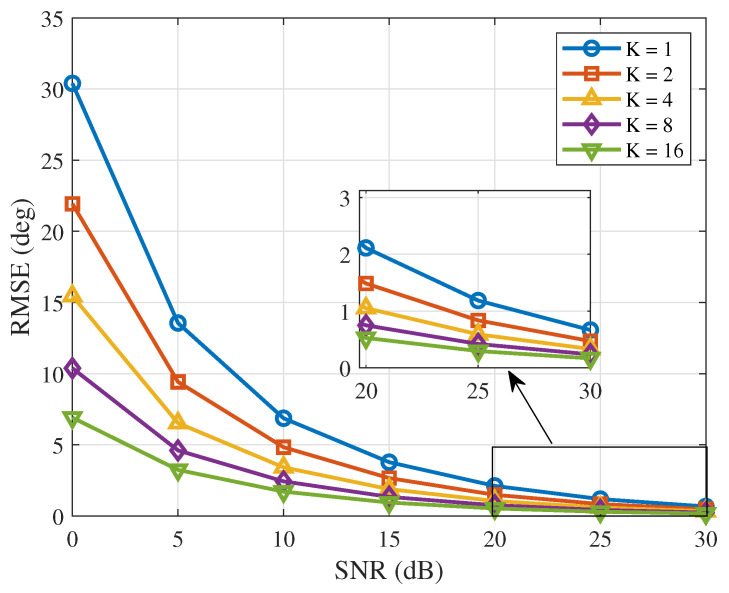
RMSE versus SNR (*K* = 1, 2, 4, 8 and 16).

After fixing the iteration step size (μ = 0.25), when the number of snapshots is set to 1, 2, 4, 8 and 16, respectively, the variation in the RMSE of the proposed algorithm with SNR is shown in Figure 3. The simulation shows that, as the SNR increases, the RMSE of the algorithm under different snapshot numbers gradually decreases, indicating that increasing the signal-to-noise ratio helps improve the phase calibration accuracy; meanwhile, as the number of snapshots increases, the RMSE under the same SNR environment is further reduced, indicating that increasing the number of snapshots can effectively suppress the influence of random noise on the measurement results. Under low-SNR conditions, increasing the number of snapshots is more effective in improving the robustness of the algorithm. The simulation results are consistent with the theoretical analysis.

### 4.2. Convergence Characteristics

**Figure 4:**
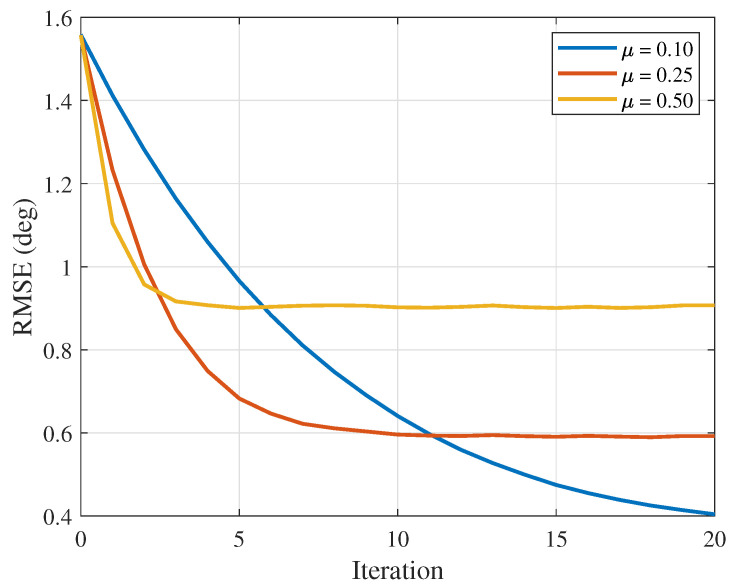
RMSE versus the number of iterations (μ = 0.1, 0.25 and 0.5).

After fixing the SNR and the number of snapshots (SNR = 25 dB, *K* = 4), the variation in the RMSE of the proposed algorithm with the number of iterations when the iteration step size is set to 0.1, 0.25 and 0.5 is shown in Figure 4. The simulation shows that, as the number of iterations increases, the RMSE of the proposed algorithm gradually decreases. Since each observation contains random errors, a smaller μ leads to a smaller update magnitude. Although this is beneficial for suppressing noise disturbance, the number of iterations required to reach stability will increase. When μ takes a larger value, noise disturbance will be aggravated. For example, when μ is 0.5, the system can reach stability in fewer iterations, but its steady-state error is significantly higher than that when μ is 0.1. The simulation results are consistent with the theoretical analysis.

**Figure 5:**
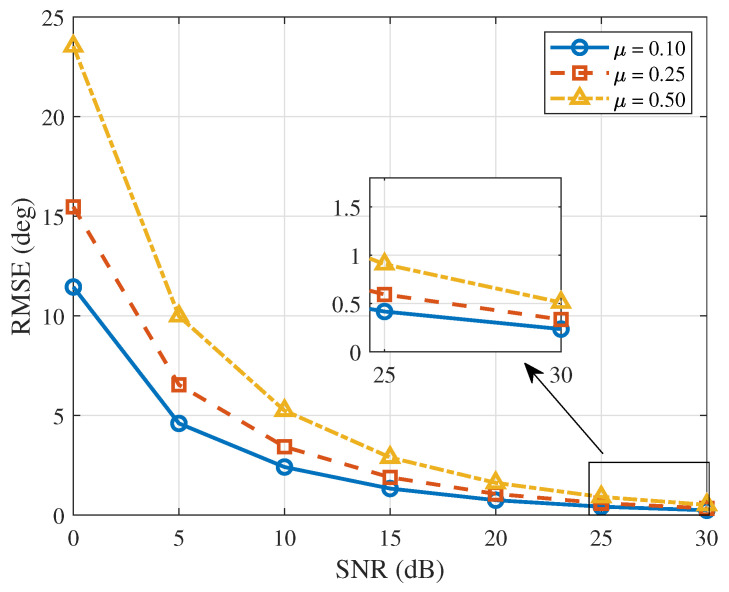
RMSE versus SNR (μ = 0.1, 0.25 and 0.5).

After fixing the number of snapshots (*K* = 4), when the iteration step size is set to 0.1, 0.25 and 0.5, respectively, the variation in the RMSE of the proposed algorithm with SNR is shown in Figure 5. The simulation shows that, as the SNR increases, the RMSE of the algorithm gradually decreases. Under the same SNR condition, the RMSE increases as μ increases, which indicates that under high-SNR conditions, the iteration step size can be appropriately increased to shorten the initial phase calibration time; under low-SNR conditions, a smaller step size should be adopted to weaken noise disturbance and improve calibration accuracy. The simulation results are consistent with the theoretical analysis.

### 4.3. Computation Time Analysis

As shown in Table 2, under the conditions of K=16, μ=0.25, and SNR =15 dB, both the average single-iteration processing time and the average total processing time increase with the array size, and the average number of iterations also shows an increasing trend. This is because, as the array size increases, the number of elements involved in each processing round also increases, which leads to an overall rise in the computational load of each iteration. Meanwhile, under noisy conditions, a larger array generally requires more iterations to satisfy the phase convergence criterion. Nevertheless, when the array size reaches 32×32, the average total processing time of the proposed algorithm remains at the millisecond level.

### 4.4. Verification of Beamforming Performance

This section further verifies the proposed method from the perspective of the array radiation pattern. Three metrics, namely beam pointing error, main-lobe peak gain, and side-lobe level, are used to quantitatively evaluate the beamforming performance before and after calibration.

#### 4.4.1. Beam Pointing Error

Let the amplitude weight of the (m,n)th array element be denoted by $a_{m,n}$, and its phase be denoted by $\phi_{m,n}$. Then, the array factor in the direction (θ,φ) can be expressed as follows:

(27) $$AF\left(\theta ,\varphi \right)=\sum_{m=0}^{M-1}\sum_{n=0}^{N-1}a_{m,n}\;e^{j\phi_{m,n}}\;e^{-jk_{0}\left(md_{1}u+nd_{2}v\right)}.$$

The corresponding amplitude of the radiation pattern is given by:

(28) $$AF_{\mathrm{amp}}\left(\theta ,\varphi \right)=\left|AF(\theta ,\varphi )\right|.$$

To determine the main-lobe direction, the global peak point is searched in the calibrated amplitude pattern, and this point is defined as the main-lobe direction.

#### 4.4.2. Side-Lobe Level

In addition to the main-lobe direction and the main-lobe peak, the side-lobe level is also an important metric for evaluating beam quality. A high side-lobe level leads to increased energy leakage in undesired directions. In this paper, the main-lobe peak position and its corresponding peak amplitude are first obtained from the pattern amplitude.

(29) $$AF_{\mathrm{amp}}\left(\theta ,\varphi \right)=\left|AF(\theta ,\varphi )\right|.$$

Then, the maximum amplitude searched in the remaining region outside the main lobe is defined as the highest side-lobe amplitude, denoted by $A_{\mathrm{sll}}$, while the main-lobe peak amplitude is denoted by $A_{\mathrm{peak}}$.

Therefore, the side-lobe level is defined as the decibel value of the highest side-lobe amplitude relative to the main-lobe peak amplitude, namely:

(30) $$\mathrm{SLL}=20\log_{10}\left(\frac{A_{\mathrm{sll}}}{A_{\mathrm{peak}}}\right).$$

For the patterns before and after calibration, the side-lobe levels are denoted by ${\mathrm{SLL}}^{\mathrm{before}}$ and ${\mathrm{SLL}}^{\mathrm{after}}$, respectively. The side-lobe improvement is further defined as follows:

(31) $$\Delta \mathrm{SLL}={\mathrm{SLL}}^{\mathrm{before}}-{\mathrm{SLL}}^{\mathrm{after}}.$$

When ΔSLL>0, it indicates that the highest side lobe is suppressed after calibration, and the side-lobe performance of the array is improved.

**Figure 6:**
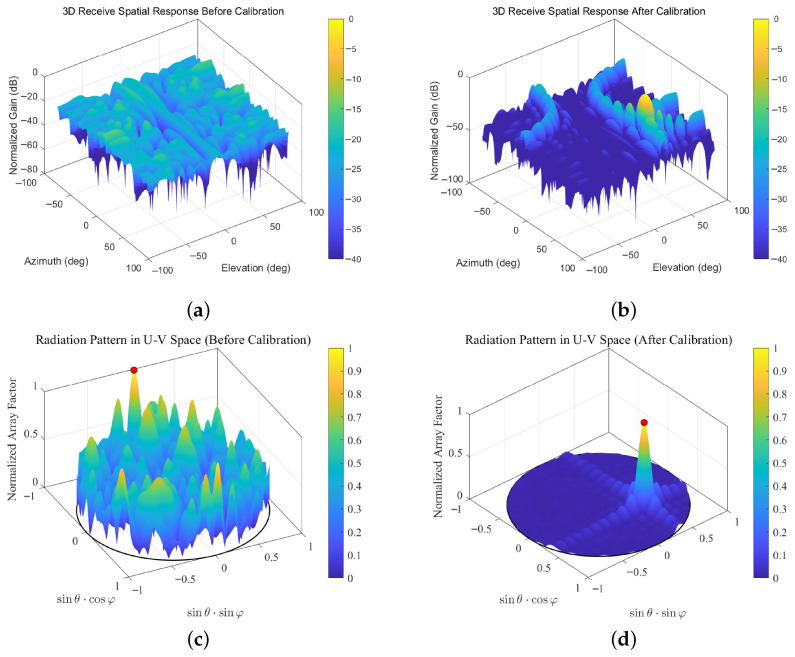
Comparison of array beam patterns before and after calibration. (**a**) 3D spatial response before calibration; (**b**) 3D spatial response after calibration; (**c**) radiation pattern in U-V space before calibration; (**d**) radiation pattern in U-V space after calibration.

As shown in Figure 6, before calibration, the array beam pattern exhibits an unfocused main lobe and relatively high side-lobes, indicating that the initial phase errors of the array elements have a significant adverse effect on the beam pointing capability of the array. After calibration, the main lobe becomes sharper and is accurately aligned with the target direction, while the side-lobe level is noticeably reduced and the energy leakage in undesired directions is effectively suppressed. This further confirms the effectiveness of the proposed method in enhancing the beamforming performance of the array.

Table 3 compares the phased-array performance metrics before and after calibration. As shown in the table, before calibration, the array beam is severely distorted by the initial phase errors, with the main-lobe pointing direction deviating to (−1.50°, −78.50°). After the proposed calibration method is applied, the beam is effectively corrected and accurately steered toward the desired direction (45.00°, 30.00°). In addition, the main-lobe gain is increased by 14.69 dB, while the side-lobe level is suppressed by 12.73 dB.

In summary, the error of the initial phase calibration decreases as the SNR increases. In practical applications, the system can achieve a trade-off between calibration accuracy and calibration efficiency by adjusting the number of snapshots and the iterative step size μ. Specifically, in low-SNR environments, the system can increase the number of sampling snapshots to reduce the impact of noise. In high-SNR environments, the number of snapshots can be reduced to improve calibration speed and decrease computational load. Meanwhile, the iterative step size μ has a significant effect on the steady-state performance. In low-SNR environments, a larger iterative step size introduces more noise into the update process, leading to a larger steady-state error. In high-SNR environments, the influence of different iterative step sizes on the steady-state error becomes smaller. This indicates that a smaller step size should be adopted in low-SNR environments to ensure calibration accuracy, whereas a larger step size can be used in high-SNR environments to accelerate convergence. In terms of computational time, as the array size increases, the average digital processing time also increases. However, even for a 32×32 array, the average total processing time of the algorithm remains at the millisecond level, demonstrating that the proposed method can meet the real-time requirement of large-scale array calibration. In addition, from the beam synthesis results, after calibration, the array main lobe can point more accurately toward the target direction, the peak main-lobe gain is improved, and the side-lobe level is effectively suppressed.

## 5. Conclusions

In summary, this paper proposes a closed-loop iterative automatic initial phase calibration method based on complex readback data of array elements. The results show that the proposed method can effectively eliminate the initial phase errors of array elements, improve the beam pointing performance, enhance the main-lobe gain, and suppress the side-lobe level while maintaining millisecond-level digital processing time for large-scale arrays. Compared with traditional calibration methods, the proposed algorithm does not require element-by-element phase adjustment or probe-based mechanical movement. Instead, it realizes automatic digital-domain calibration through a closed-loop iterative update mechanism for the phase compensation table, enabling fast and automatic phased-array calibration. Owing to its good robustness and adaptability, the proposed method has broad application prospects in satellite Internet, 6G, and low-altitude economy networks.

## Figures and Tables

**Table 1:** Comparison of the proposed method with SPGD and deep -earning-based calibration methods.

Method	Calibration Strategy	Results	Advantages	Limitations
SPGD Method	OTA power-feedback iteration	For a 16-element array, approximately 50 iterations and 100 amplitude measurements are required	No element-by-element scanning is required; full-array joint calibration can be achieved; calibration accuracy is comparable to that of the REV method	The number of iterations increases significantly as the number of elements grows
Deep Learning Method	HFSS full-wave simulation + neural network inference	Approximately 8000 HFSS simulations are used to construct the dataset; for a 4×4 microstrip patch array with a 6-bit BFIC, the 1D-CNN can achieve a beam pointing result of 44.8° at a target angle of 45°	Fast online inference; capable of learning complex non-ideal effects such as mutual coupling, quantization errors, and edge effects; suitable for rapid compensation on fixed platforms	Requires large-scale offline datasets and full-wave simulations
Proposed Method	Closed-loop iterative update based on element-level complex readback	For a 256-element array, the digital computation time is about 1.6 ms. For a 256-element array, the digital computation time is about 1.6 ms; the main-lobe gain is improved by 14.69 dB, and the side-lobe level is reduced by 12.72 dB.	Provides a clear update direction; can directly estimate the relative phase residual using element-level complex information; suitable for fast online array calibration	Depends on the direction and frequency information of the calibration source

**Table 2:** Average post-processing time statistics of the proposed algorithm under different array sizes.

Array Size (M×N)	Avg. Iterations	Avg. Single-Iteration Time (ms)	Avg. Total Time (ms)
4×4	13.95	0.01044	0.17101
8×8	17.87	0.021417	0.44404
16×16	18.53	0.077087	1.6123
32×32	19.48	0.12987	2.7268
64×64	19.74	1.2282	25.411

**Table 3:** Comparison of beamforming performance metrics before and after calibration.

Performance Metric	Before Calibration	After Calibration
Direction	(−1.50°, −78.50°)	(45.00°, 30.00°)
Beam Pointing Error $\theta_{\mathrm{err}}$	111.78°	≈0.00°
Normalized Peak Gain $G_{\mathrm{peak}}$	−14.69 dB	0.00 dB
Peak Side-Lobe Level (SLL)	−0.32 dB	−13.05 dB

## Data Availability

Data are contained within this article.
